# Supplementary material for: Structural and biochemical mechanism for increased infectivity and immune evasion of Omicron BA.2 variant compared to BA.1 and their possible mouse origins
Source: Cell Res. 2022 May 31;32(7):609–20. doi: 10.1038/s41422-022-00672-4 (PMC9152305; doi:10.1038/s41422-022-00672-4)
Supplement: Supplementary file 1 — Supplementary information, Fig. S1 [file 41422_2022_672_MOESM1_ESM.pdf]

Supplementary information, Fig. S1

a

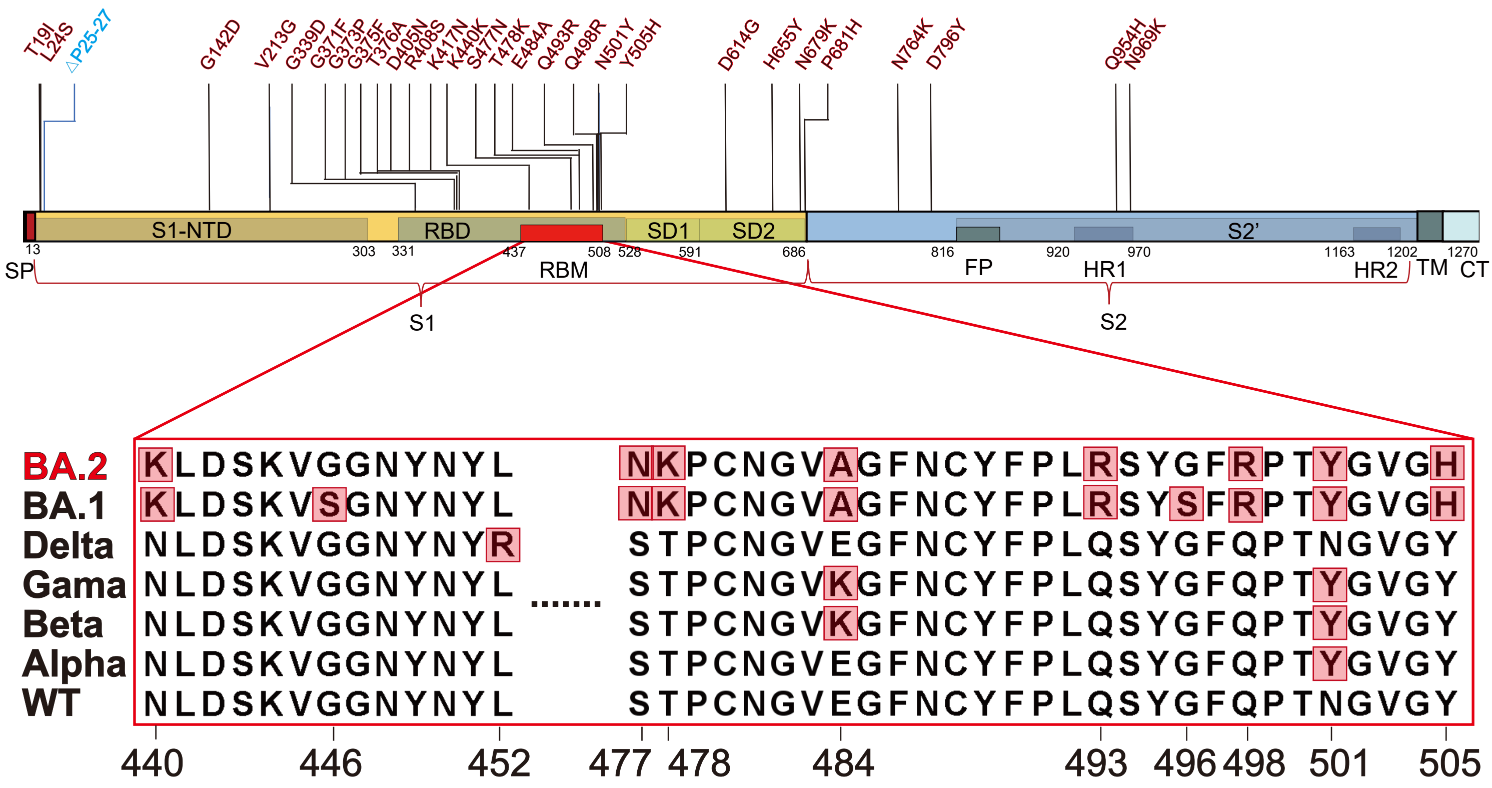

Fig. S1. Schematic of the Omicron BA.2 spike protein domain architecture.

(a) Schematic of the Omicron BA.2 spike protein domain architecture. Mutations of the Omicron spike protein are labeled with different colors (blue for deleting mutation, brown for inserting mutation). Mutations in RBM are compared with WT SARS-CoV-2 and five other VOC strains. SP, signal peptide; RBM, receptor-binding motif; SD1, subdomain 1; SD2, subdomain 2; FP, fusion peptide; HR1, heptad repeat 1; HR2, heptad repeat 2; TM, transmembrane region; CT, cytoplasmic tail.
