# Supplementary material for: Structural and biochemical mechanism for increased infectivity and immune evasion of Omicron BA.2 variant compared to BA.1 and their possible mouse origins
Source: Cell Res. 2022 May 31;32(7):609–20. doi: 10.1038/s41422-022-00672-4 (PMC9152305; doi:10.1038/s41422-022-00672-4)
Supplement: Supplementary file 2 — Supplementary information, Fig. S2 [file 41422_2022_672_MOESM2_ESM.pdf]

Supplementary information, Fig. S2

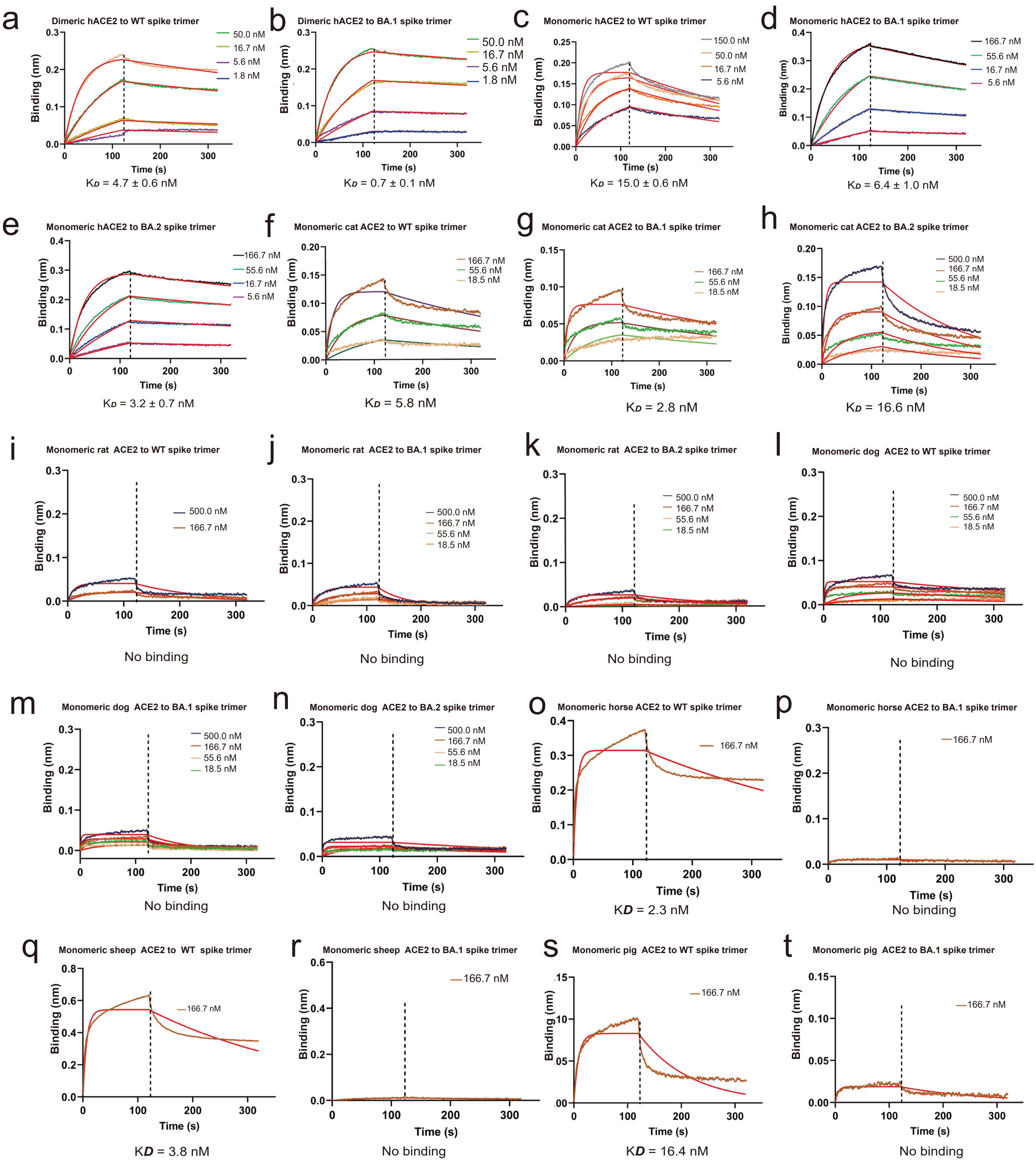

Fig. S2. Binding affinities of ACE2 from different species to spike protein from different strains

(a and b) Binding of dimeric hACE2 to WT (a) and BA.1 (b) spike trimer; (c-e) binding of monomeric human ACE2 to WT (c), BA.1 (d) and BA.2 (e) spike trimer; (f-h) binding of monomeric cat ACE2 to WT (f), BA.1 (g) and BA.2 (h) spike trimer; (i-k) binding of monomeric rat ACE2 to WT (i), BA.1 (j) and BA.2 (k) spike trimer; (l-n) binding of monomeric dog ACE2 to WT (l), BA.1 (m) and BA.2 (n) spike trimer; (o and p) binding of monomeric horse ACE2 to WT (o) and BA.1 (p) spike trimer; (q and r) binding of monomeric sheep ACE2 to WT (q) and BA.1 (r) spike trimer; (s and t) binding of monomeric pig ACE2 to WT (s) and BA.1 (t) spike trimer. These data are determined by BLI. The KD values are further determined with Octet Data Analysis HT 11.0 software using a 1:1 global fit model.
