# Supplementary material for: Structural and biochemical mechanism for increased infectivity and immune evasion of Omicron BA.2 variant compared to BA.1 and their possible mouse origins
Source: Cell Res. 2022 May 31;32(7):609–20. doi: 10.1038/s41422-022-00672-4 (PMC9152305; doi:10.1038/s41422-022-00672-4)
Supplement: Supplementary file 3 — Supplementary information, Fig. S3 [file 41422_2022_672_MOESM3_ESM.pdf]

Supplementary information, Fig. S3

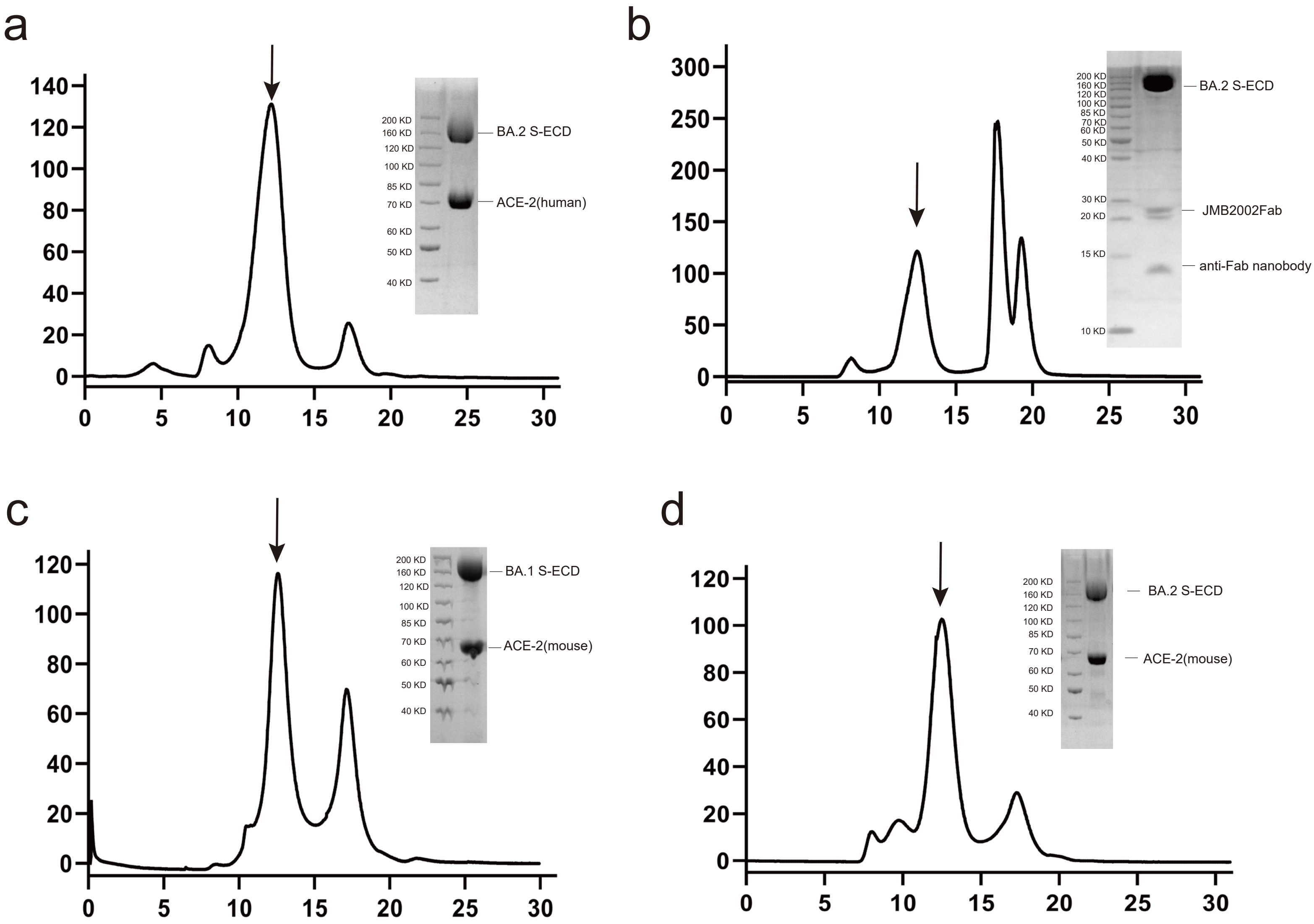

Fig. S3. Purification and characterization of the Omicron spike protein complex.
(a-d) Gel filtration profile of the Omicron BA.2 ECD-hACE2 complex (a), Omicron BA.2 ECD-Fab complex (b), Omicron BA.1 ECD-mACE2 complex (c), Omicron BA.2 ECD-mACE2 complex (d), all showing a sharp peak, and corresponding SDS gel showing balanced ratios for each subunit.
