# Supplementary material for: Structural and biochemical mechanism for increased infectivity and immune evasion of Omicron BA.2 variant compared to BA.1 and their possible mouse origins
Source: Cell Res. 2022 May 31;32(7):609–20. doi: 10.1038/s41422-022-00672-4 (PMC9152305; doi:10.1038/s41422-022-00672-4)
Supplement: Supplementary file 5 — Supplementary information, Fig. S5 [file 41422_2022_672_MOESM5_ESM.pdf]

Supplementary information, Fig. S5

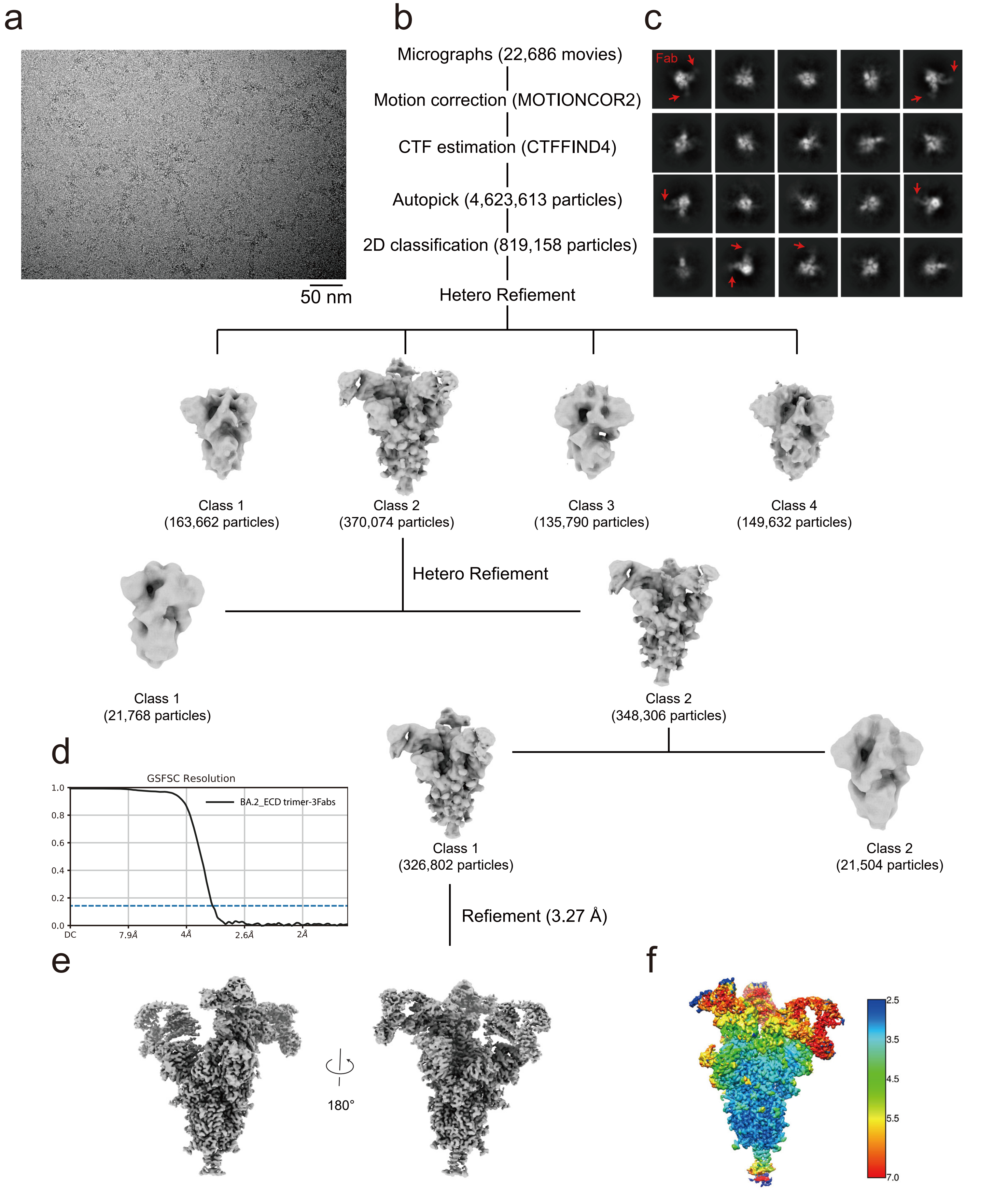

Fig. S5. Cryo-EM data processing of the Omicron BA.2 ECD-Fab complex.

(a) A representative cryo-EM micrograph of Omicron BA.2 ECD-Fab complex with 50 nm scale bar included as a size reference. (b) Computational processing of cryo-EM data. (c) Twenty representative reference-free two-dimensional (2D) cryo-EM class averages reveal the Fab density (red arrow). (d)The FSC curves for the reconstructions.(e) The EM maps of Omicron BA.2 ECD trimer-3Fabs in two front views. (f) Local resolution of sub-reconstructions of BA.2 ECD trimer-3Fabs (left panel) and resolution bar is shown in the right panel.
