# Supplementary material for: Structural and biochemical mechanism for increased infectivity and immune evasion of Omicron BA.2 variant compared to BA.1 and their possible mouse origins
Source: Cell Res. 2022 May 31;32(7):609–20. doi: 10.1038/s41422-022-00672-4 (PMC9152305; doi:10.1038/s41422-022-00672-4)
Supplement: Supplementary file 6 — Supplementary information, Fig. S6 [file 41422_2022_672_MOESM6_ESM.pdf]

Supplementary information, Fig. S6

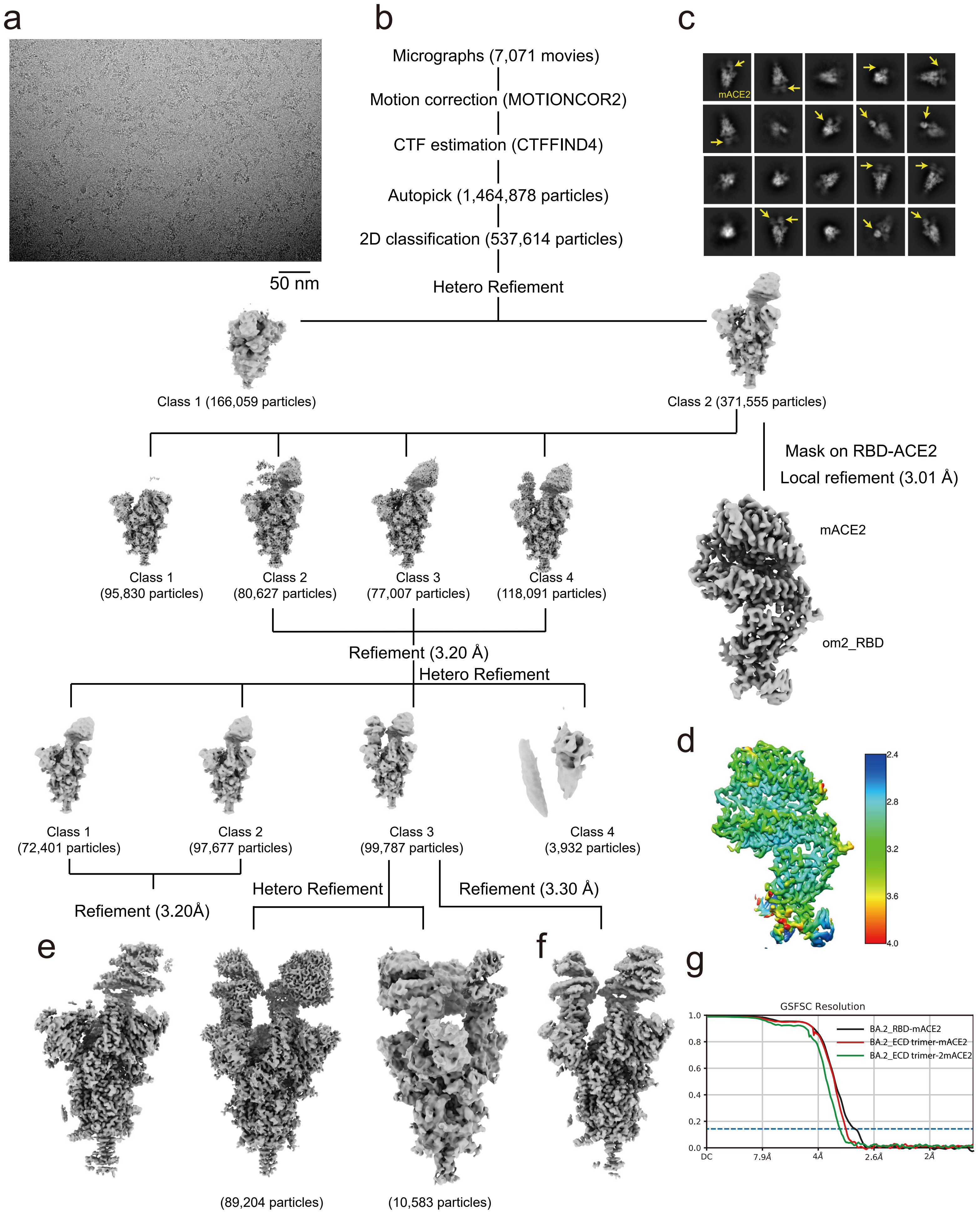

Fig. S6 Cryo-EM data processing of the Omicron BA.2 spike protein-mACE2 complex.

(a) A representative cryo-EM micrograph of Omicron BA.2 ECD-mACE2 complex with 50 nm scale bar included as a size reference. (b) Computational processing of cryo-EM data. (c) Twenty representative reference-free two-dimensional (2D) cryo-EM class averages reveal the mACE2 density (yellow arrow). (d) Local resolution of sub-reconstructions of RBD-mACE2 (left panel) and resolution bar is shown in the right panel. (e-f) The EM maps of Omicron BA.2 ECD trimer bound to 1 mACE2 (e), 2 mACE2 (f). (g)The FSC curves for the reconstructions. Color scheme: BA.2 RBD-mACE2, black; BA.2 ECD trimer-mACE2, red; BA.2 ECD-trimer-2mACE2, green. The resolution of the reconstructions using the Fourier shell cutoff at 0.143 is shown.
